# The Scissors Effect of the Macromolecular Crosslinker on the Glass Transition of Polystyrene in Its Conetworks with Poly(dimethylsiloxane)

**DOI:** 10.3390/polym17121656

**Published:** 2025-06-14

**Authors:** Anna Petróczy, István Szanka, Laura Bereczki, Nóra Hegyesi, János Madarász, Béla Iván

**Affiliations:** 1 Polymer Chemistry and Physics Research Group, Institute of Materials and Environmental Chemistry, HUN-REN Research Centre for Natural Sciences, Magyar tudósok körútja 2, H-1117 Budapest, Hungary; 2 George Hevesy PhD School of Chemistry, Institute of Chemistry, Faculty of Science, Eötvös Loránd University, Pázmány Péter sétány 2, H-1117 Budapest, Hungary; 3 Chemical Crystallography Research Laboratory, Centre of Structural Science, HUN-REN Research Centre for Natural Sciences, Magyar tudósok körútja 2, H-1117 Budapest, Hungary; 4 Department of Physical Chemistry and Materials Science, Budapest University of Technology and Economics, Műegyetem rkp. 3, H-1111 Budapest, Hungary; 5 Department of Inorganic and Analytical Chemistry, Budapest University of Technology and Economics, Műegyetem rkp. 3, H-1111 Budapest, Hungary

**Keywords:** polymer conetworks, polystyrene, poly(dimethylsiloxane), divinylbenzene, glass transition temperature, *M*_c_, Fox–Flory equation, scissors effect

## Abstract

The glass transition temperature (*T*_g_) is undoubtedly one of the most important characteristics of polymers, and investigating its dependence on their structure and composition is crucial from both fundamental and application points of view. This study deals with the unexpected relationship between *T*_g_ and the average molecular weight between crosslinking points (*M*_c_) in nanophase-separated polystyrene-*l*-poly(dimethylsiloxane) (PSt-*l*-PDMS) and polystyrene-*l*-poly(dimethylsiloxane)/divinylbenzene (PSt-*l*-PDMS/DVB) polymer conetworks. In order to reveal the correlation between the *T*_g_ and *M*_c_, a library of PSt-*l*-PDMS and PSt-*l*-PDMS/DVB conetworks was synthesized, and their compositions and *T*_g_s were determined. Instead of the expected increase of *T*_g_ with decreasing *M*_c_, a reverse correlation was found. Namely, the *T*_g_ decreases with decreasing *M*_c_ in these conetworks. Correlation analyses showed that the *T*_g_ linearly depends on 1/*M*_c_, similar to the Fox–Flory relationship for homopolymers with their *M*_n_, that is, *T*_g_ = *T*_g,ꝏ_ −*K*/*M*_c_ for the investigated conetworks, independent of the absence or presence of relatively low amounts of DVB as an additional small molecular weight crosslinker. This means that the PDMS macrocrosslinker acts like scissors by interrupting the mobility of the crosslinked PSt chains in the conetworks, and the *T*_g_ of the PSt segments will be close to that of PSt homopolymers with the same *M*_n_ as *M*_c_, as found by comparison. Consistent with previous findings with other conetworks, the presence of the *scissors effect* of the macromolecular crosslinker in the PSt-*l*-PDMS and PSt-*l*-PDMS/DVB conetworks indicates that the scissors effect is a general phenomenon for polymer conetworks formed by crosslinking with a macromolecular crosslinker. The observed unusual *T*_g_ versus *M*_c_ relationship in the conetworks can be utilized in designing such novel materials with predetermined *T*_g_s required for targeted applications.

## 1. Introduction

Beyond a doubt, the glass transition temperature (*T*_g_) is one of the most important characteristics of polymers. Since the *T*_g_ is the temperature at which a polymer undergoes a transition from a glassy state to an elastic (viscoelastic) state, which results in substantial changes in its physical properties, such as elasticity, strength, and stiffness, it is a critical factor for both polymer processing and applications. As a consequence, the effect of structure, topology (i.e., linear, branched, and crosslinked) and the molecular weight of macromolecules on their *T*_g_ has been widely investigated since the beginning of polymer science, technology, and production. One of the recently emerging classes of crosslinked polymers belongs to conetworks, which are composed of different polymer chains coupled with chemical bonds to each other (see e.g., Refs. [1,2,3,4,5,6,7,8,9,10,11,12,13,14,15,16,17,18,19,20,21,22,23,24,25,26,27,28,29,30,31,32,33,34,35,36,37,38,39,40,41,42,43,44,45,46,47,48,49,50,51,52,53,54,55,56,57,58,59,60,61,62,63,64,65,66,67,68,69,70,71,72,73] and references therein). In these macromolecular assemblies, two or more polymer chains, which are mostly immiscible, are crosslinked either by covalent, ionic, or supramolecular bonds. Due to the mobility restriction of the crosslinked macromolecules, conetworks with nanophasic morphology are obtained in the cases of immiscible polymer components [13,14,15,16,27,28,51,52,53,61,68,73]. These conetworks usually have a bicontinuous (cocontinuous) structure with a broad composition window [13,14,28,53,61,73]. Due to the unique bicontinuous nanophase separated structure and component-selective swelling behavior of polymer conetworks, these materials offer advanced applications in various fields, such as sensors [5,6,7], self-healing materials [10,11,40,60], membranes [24,25,26], drug delivery [33,34,35,61,62,63], ophthalmic devices [45,46], catalyst supports [47,48,49], etc.

Although the glass transition temperature is a critical property of the amorphous macromolecular materials, not only from fundamental aspects but also from an application point of view, surprisingly, relatively few reports have dealt with the thermal transition behavior of the polymeric components in conetworks, i.e., *T*_g_ and/or crystallization and melting temperatures [12,13,50,51,52,53,54,55,56,57,58,59,71,72,73]. Unexpectedly, in two cases, that is, for the poly(N-vinylimidazole)-*l*-poly(tetrahydrofuran) (PVIm-*l*-PTHF) [12] and poly(methyl methacrylate)-*l*-polyisobutylene (PMMA-*l*-PIB) [13] conetworks, a Fox–Flory-type correlation [74] was found between the *T*_g_ of the crosslinked polymers, i.e., PVIm and PMMA, and the average molecular weight between crosslinking points (*M*_c_). This means that the *T*_g_ decreases with decreasing *M*_c_, that is, by increasing crosslinking density, according to the Fox–Flory equation for the *T*_g_ dependence of homopolymers on the number average molecular weight (*M*_n_) [74]:

(1) $$T_{g}=T_{g,\infty }-\frac{K}{M_{n}}$$

where *T*_g,ꝏ_, represents the glass transition temperature of the polymer with infinite molecular weight, and *K* is a constant. In the case of the mentioned examples [12,13], this correlation is valid by substituting *M*_n_ with *M*_c_. Furthermore, it was found for the PMMA-*l*-PIB conetworks that the *K* value fits well with that of the PMMA homopolymers in the same molecular weight region [13]. These findings fully contradict expectations, because it is well known that increasing the crosslinking density leads to increasing the *T*_g_ in polymer networks crosslinked with crosslinkers of low molecular weights [75,76,77], on the one hand. On the other hand, the observed decrease in *T*_g_ with increasing crosslinking density, i.e., with decreasing *M*_c_, indicates that the macromolecular crosslinkers have a ‘*scissors effect*’ in polymer conetworks. As a consequence, in relation to their glass transition, the polymer segments between the crosslinking junctions behave like the corresponding homopolymers with molecular weights similar to the *M*_c_ in the conetworks [12,13].

Recently, we reported on the synthesis, structural analysis, and selective swelling behavior of nanophase-separated conetworks of polystyrene (PSt) with poly(dimethylsiloxane) (PDMS) as a macrocrosslinker, both in the absence and presence of divinylbenzene (DVB) as an additional low molecular weight crosslinker [73]. In the examined cases, it was found that the *T*_g_ of the crosslinked PSt component was lower than expected, while the *T*_g_ of the PDMS is nearly the same as that of the PDMS homopolymers, i.e., in the range of −120 °C [78,79,80,81,82,83]. On the other hand, the *T*_g_s of polystyrene homopolymers depend strongly on molecular weight below ~20 kDa [84,85,86,87], then level off above this molecular weight with *T*_g_ in the 100–110 °C range, and it follows the Fox–Flory relationship [86,88]. It must also be mentioned that the *T*_g_ of the components in PSt-PDMS block copolymers is the same as that of the components, due to the immiscibility of PSt and PDMS [89,90].

Motivated by the unexpected glass transition behavior of the PSt component in the novel polystyrene-*l*-poly(dimethylsiloxane) (PSt-*l*-PDMS) and polystyrene-*l*-poly(dimethylsiloxane)/divinylbenzene (PSt-*l*-PDMS/DVB) conetworks (-*l*- stands for *linked by*), a library of such conetworks with varying compositions and PDMS with different molecular weights has been prepared in order to investigate the effect of crosslinkers on the *T*_g_ of the crosslinked PSt. Herein, we report on the results of the systematic studies on the thermal transitions of the PSt and PDMS components in the PSt-*l*-PDMS and PSt-*l*-PDMS/DVB conetworks as determined by DSC measurements. Then, a subsequent correlation analysis was performed to determine the existence of the scissors effect by the PDMS crosslinker in these conetworks, and to examine the effect of the DVB, as an additional low molecular weight crosslinker, on the *T*_g_ of the crosslinked PSt in the PSt-*l*-PDMS/DVB conetworks.

## 2. Materials and Methods

### 2.1. Materials

Styrene (99%, Sigma-Aldrich, Steinheim, Germany) and divinylbenzene (DVB) (80% m/p-divinylbenzene and 20% ethylstyrene, Honeywell Fluka, Steinheim, Germany) were distilled under vacuum from calcium hydride before use. Methacryloxypropyl-telechelic poly(dimethylsiloxane)s (MA-PDMS-MA, *M*_n,PDMS_ = 4700, 9000 and 22,200 Da; Gelest Inc., Morrisville, PA, USA) were purified to remove the inhibitor by precipitation from hexane solution to methanol, then redissolved and extracted with distilled water, dried on anhydrous magnesium sulfate, and finally, the hexane was removed under reduced pressure followed by drying to constant weight under vacuum at room temperature. The initiator, α,α’-azobisisobutyronitrile (AIBN, Sigma-Aldrich, Steinheim, Germany) was crystallized twice in methanol, filtered, and dried under vacuum at room temperature prior to use. Tetrahydrofuran (THF) (VWR International Ltd., Debrecen, Hungary) and toluene (VWR International Ltd., Debrecen, Hungary) for conetwork syntheses were distilled before use. Other solvents, including hexane (Molar Chemicals, Halásztelek, Hungary), 1-nitropropane (Sigma-Aldrich, Steinheim, Germany), and methanol (VWR International Ltd., Debrecen, Hungary) were used as received.

### 2.2. The Synthesis of PSt-l-PDMS and PSt-l-PDMS/DVB Conetworks, and Polystyrene Homopolymer

The PSt-*l*-PDMS and PSt-*l*-PDMS/DVB conetworks were synthesized via free radical copolymerization of MA-PDMS-MA, styrene, and divinylbenzene as previously described [73]. Briefly, the conetworks were synthesized with MA-PDMS-MA contents varying in the 30–80 wt% range. The PSt-*l*-PDMS/DVB conetworks were prepared with an St/DVB weight ratio of 36:1. The predetermined amounts of the reactants were measured and mixed in a glovebox under nitrogen atmosphere and dissolved in THF or toluene (for conetwork syntheses with PDMS containing *M*_n,PDMS_ of 9 or 22.2 kDa) to obtain total reactant concentration of 0.4 g/mL. The [M]/[I]^0.5^ ratio was 23.2 in all cases, where [M] and [I] stand for the total concentrations of the monomers and AIBN initiator, respectively. The homogenized reaction mixtures were poured into Teflon molds, sealed, and reacted at 60 °C for 72 h. After the synthesis, the samples were dried and sequentially extracted with hexane and 1-nitropropane to remove unreacted PDMS, and styrene and derivatives, respectively. The PSt homopolymer (*M*_n,PSt_ = 44 kDa, *D* = 3.08) was synthesized under the same conditions as the conetworks.

The conetwork samples are identified with their components (S for styrene, D for divinylbenzene, followed by 36, i.e., the St/DVB weight ratio when DVB was used), the *M*_n_/1000 of the MA-PDMS-MA macrocrosslinker, and the PDMS contents of the conetworks, which were determined by either elemental analysis or the composition of the extracted materials (see Ref. [73] for details).

### 2.3. Differential Scanning Calorimetry (DSC)

The DSC measurements were carried out on Mettler Toledo DSC821e (Mettler Toledo, Greifensee, Switzerland) equipment in the temperature range of 0 to 200 °C with 10 °C/min heating/cooling rate under 80 mL/min nitrogen flow. The low temperature DSC measurements from −150 °C to zero degree were performed on a Perkin Elmer Diamond DSC (Perkin Elmer, Norwalk, CT, USA) with 10 °C/min heating/cooling rate under 20 mL/min helium flow. Furthermore, TA Instruments 2920 Modulated DSC (New Castle, DE, USA) equipment was also used in this temperature range (−150 to 0 °C). The inflection points of the glass transitions in the second heating runs were determined as the glass transition temperatures (*T*_g_).

## 3. Results and Discussion

**Scheme 1:**
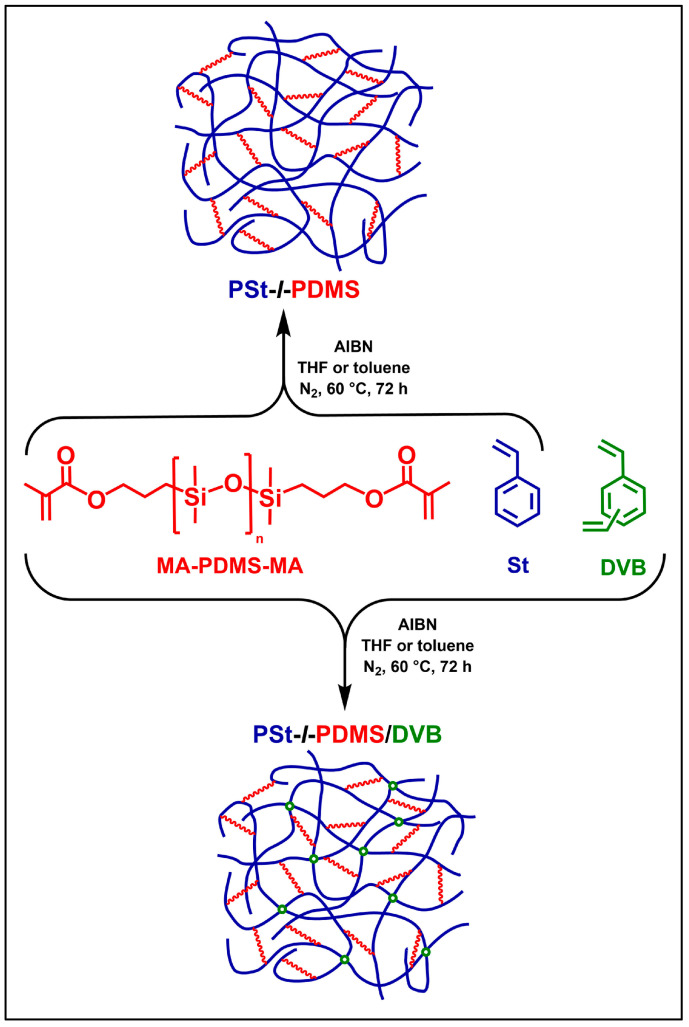
The reaction route for the syntheses of polystyrene-*l*-poly(dimethylsiloxane) (PSt-*l*-PDMS) and polystyrene-*l*-(poly(dimethylsiloxane)/divinylbenzene) (PSt-*l*-PDMS/DVB) conetworks.

As previously reported, the PSt-*l*-PDMS and PSt-*l*-PDMS/DVB conetworks possess bicontinuous nanophase-separated morphology with distinct polystyrene and poly(dimethylsiloxane) nanodomains, as determined by small angle X-ray scattering (SAXS) and atomic force microscopy (AFM) measurements [73]. For the examined cases in this study, DSC curves also indicated the phase separation in these conetworks with two separate glass transition temperatures (*T*_g_) for the PSt and the PDMS components [73]. While these measurements showed the *T*_g_ of PDMS within the range of its homopolymer, the observed *T*_g_ of PSt in the conetworks fell below that of the expected *T*_g_ of the PSt homopolymer. In order to gain insight into the glass transition behavior of PSt in the PDMS-crosslinked conetworks, and in line with previous findings on the scissors effect of macrocrosslinking in other conetworks [12,13], a library of PSt-*l*-PDMS and PSt-*l*-PDMS/DVB conetworks was synthesized and analyzed by DSC measurements. Scheme 1 shows the synthesis routes for obtaining such conetworks in the absence or presence of DVB, as an additional low molecular weight crosslinker, which is a widely used crosslinker for preparing PSt networks. As displayed in this scheme, the conetworks were prepared by free radical polymerization in a common solvent for all the components, including the resulting PSt. As shown in Table 1, this process with all the applied methacrylate-telechelic PDMS macrocrosslinkers resulted in conetworks with relatively high gel fractions after careful extraction, and their composition was close to that of the feed. The addition of DVB led to higher gel fractions, and it did not prevent the incorporation of the PDMS macrocrosslinkers.

**Figure 1:**
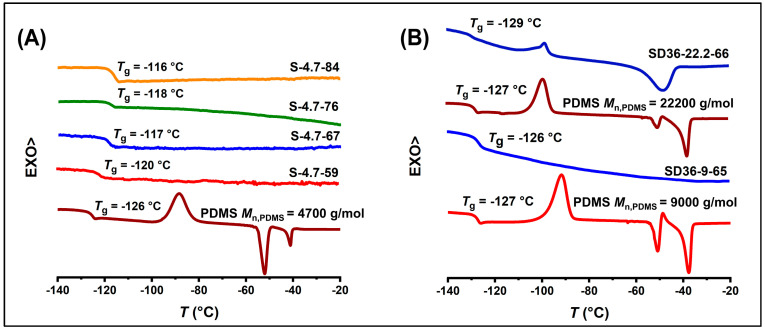
The DSC curves in the temperature range of the glass transition, crystallization, and melting of PDMS for the PDMS homopolymers, the PSt-*l*-PDMS (**A**), and PSt-*l*-PDMS/DVB (**B**) conetworks.

**Figure 2:**
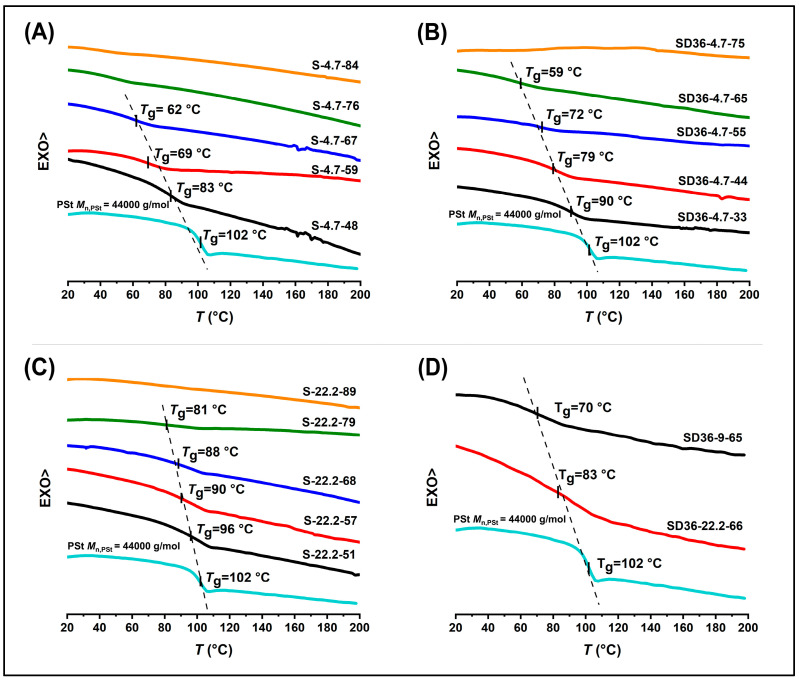
The DSC curves and glass transition temperatures of the polystyrene component in the PSt-*l*-PDMS(4.7k) (**A**), PSt-*l*-PDMS(4.7 k)/DVB (**B**), PSt-*l*-PDMS(22.2 k) (**C**), and PSt-*l*-PDMS(9 k&22.2 k)/DVB (**D**) conetworks from 0 °C to 200 °C (the dotted lines are only for guiding the eyes).

Figure 1 and Figure 2 show the DSC scans in the temperature ranges of −150–0 °C and 0–200 °C for detecting the thermal transitions of the PDMS and PSt components, respectively. These figures clearly indicate that the *T*_g_s of both components can be detected in the PSt-*l*-PDMS and PSt-*l*-PDMS/DVB conetworks. Specifically, there is one glass transition near the *T*_g_ of the PDMS homopolymers, and another one for the PSt component in the higher temperature region of the DSC measurements. Evidently, the two separate glass transitions mean microphase separation of the PSt and the PDMS domains with significant segregation in these conetworks, which is consistent with previous reported SAXS and AFM results [73]. It must be noted that the glass transition of the PSt component with sufficient certainty cannot be detected in conetworks with low PSt contents (i.e., less than ~25 wt%), as shown in Figure 2A–C. As displayed in Figure 1, the PDMS homopolymers have *T*_g_s, cold crystallization, and melting transitions in the temperature ranges as expected on the basis of literature reports [78,79,80,81,82,83,89,90]. However, signals indicating crystallization of PDMS cannot be detected in both the PSt-*l*-PDMS and PSt-*l*-PDMS/DVB conetworks with PDMS macrocrosslinkers having *M*_n,PDMS_ of 4.7 and 9 kDa. This means that crystallization of the PDMS component in these conetworks is fully prevented, which is attributed to the nanoconfined constraints of the PDMS nanodomains by the PSt nanophases in the bicontinuous nanophasic morphological structure of these conetworks [73]. Similar findings were recently reported for block copolymers of poly(poly(ethylene glycol) acrylate) (PEGA_76_) with poly(2-hydroxyethyl acrylate-polyhexanoate), that is, decrease in the crystalline fraction of PEGA76 by decreasing its fraction, but having nearly constant *T*_g_ of this component [91]. Weak cold crystallization and melting peaks appear in the DSC scan of the conetwork prepared with the MA-PDMS-MA macrocrosslinker with *M*_n,PDMS_ of 22.2 kDa (Figure 1B). This is presumably due to the higher mobility of the longer PDMS chains in this case.

As the data indicate in Figure 2 and Table 1, the *T*_g_ of PSt in the conetworks decreases with increasing PDMS macrocrosslinker content, that is, with increasing crosslinking density. This is in sharp contrast to expectations. As evidenced by the experimental data, the glass transition temperature increases with the increasing amount of the crosslinker in cases of polymer networks with low molecular weight crosslinkers [75,76,77]. Inspired by previous similar findings with conetworks, which led to the discovery of the scissors effect [12,13], the *M*_c_, i.e., the average molecular weight between crosslinking points, was determined in order to carry out a correlation analysis between the observed *T*_g_ and *M*_c_ values of the PSt-*l*-PDMS and PSt-*l*-PDMS/DVB conetworks. The *M*_c_ for the PSt-*l*-PDMS conetworks can be obtained by the following relationship:

(2) $$M_{c}=\frac{W_{St}\cdot M_{n,PDMS}}{2\cdot W_{PDMS}}$$

where *W*_St_ and *W*_PDMS_ denote the weight fractions of PSt and PDMS, respectively, and *M*_n,PDMS_ stands for the number average molecular weight of the PDMS macrocrosslinker. In the case of the PSt-*l*-PDMS/DVB conetworks, by taking into account the additional DVB crosslinker as well, the *M*_c_ can be obtained by the following formula:

(3) $$M_{c}=\frac{W_{St}}{2\cdot \left(\frac{W_{PDMS}}{M_{n,PDMS}}+\frac{W_{DVB}}{M_{DVB}}\right)},$$

where *W*_DVB_ and *M*_DVB_ denote the weight fraction and molecular weight of DVB, respectively. The *M*_c_ data obtained by these two equations fall in the range of 400–10,500 g/mol, that is, covering a more than one order of magnitude range, as presented in Table 1.

**Figure 3:**
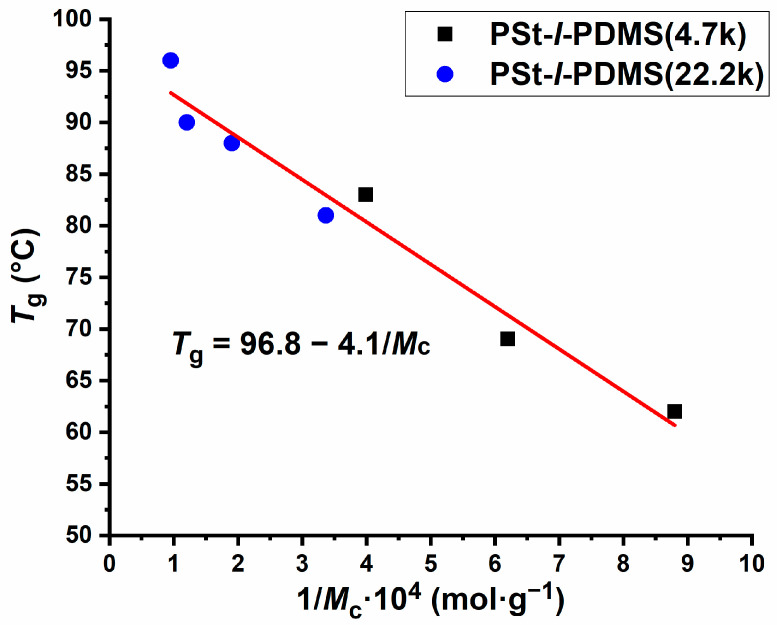
The Fox–Flory plot of the glass transition temperatures (*T*_g_) of the PSt component of the PSt-*l*-PDMS conetworks with PDMS macrocrosslinkers containing *M*_n,PDMS_ of 4.7 and 22.2 kDa as a function of 1/*M*_c_.

The *T*_g_ data for the PSt-*l*-PDMS conetworks prepared with two different MA-PDMS-MA crosslinkers having *M*_n,PDMS_ of 4.7 and 22.2 kDa are plotted as a function of 1/*M*_c_ in Figure 3. Apparently, the *T*_g_ data in this plot can be well fitted with a straight line, resulting in a slope of *K* = 4.1 × 10^−4^ °C·g/mol and an intercept of *T*_g,ꝏ_ = 96.8 °C, which agrees well with the *T*_g_ of high molecular weight PSt. This linear correlation undisputedly indicates that the *T*_g_ of PSt, crosslinked with the PDMS macrocrosslinkers, follows the Fox–Flory relationship when *M*_c_ is considered as the variable, according to the following equation:

(4) $$T_{g}=T_{g,\infty }-\frac{K}{M_{c}}$$

**Scheme 2:**
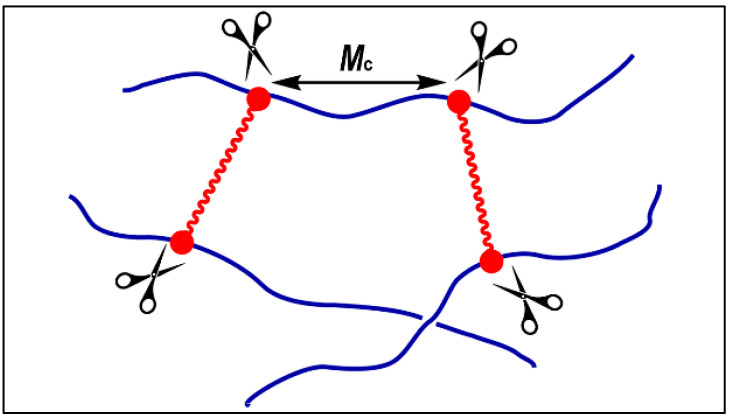
The scissors effect in the conetworks and the *M*_c_ between the two crosslinking points, (the red polymer chains represent the macromonomer crosslinker).

**Figure 4:**
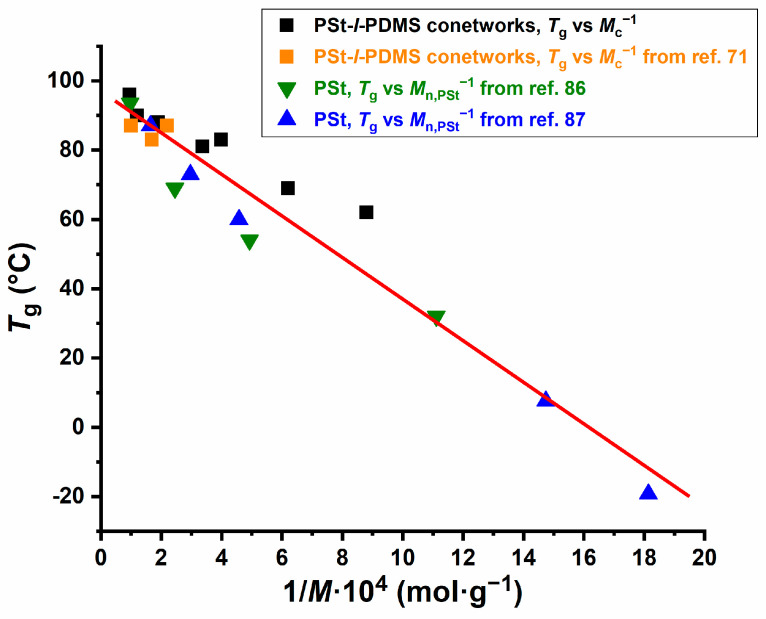
The *T*_g_ of the PSt component of PSt-*l*-PDMS conetworks (black and light brown) as a function 1/*M*_c_, and the *T*_g_ of PSt homopolymers as a function 1/*M*_n,PSt_ (green and blue). Data from Refs. [71,86,87].

This means that the polystyrene segments between two crosslinking points in the PSt-*l*-PDMS conetworks for their glass transition behave like the corresponding homopolymers with the same average molecular weight. In other words, the MA-PDMS-MA macrocrosslinkers act like scissors from the point of view of glass transition of the crosslinked PSt, as illustrated in Scheme 2. Comparing the *K* value obtained for the PSt-*l*-PDMS conetworks with that of PSt homopolymers raises some difficulties. As the literature indicates, three different regions can be distinguished in the *T*_g_ dependence of PSt on its number average molecular weight (*M*_n,PSt_) [84,85]. This includes two low molecular weight regions with varying *M*_n,PSt_ dependence without sharp boundaries, and a higher molecular weight region with nearly constant *T*_g_. Because the *M*_c_ region in the investigated PSt-*l*-PDMS conetworks lies in the borderline region of the two low molecular weight areas of the PSt homopolymers, only the *T*_g_ data of PSt homopolymers from the same molecular weight regions than that of the coneworks’ *M*_c_ can be compared. Figure 4 displays the literature *T*_g_ of PSt homopolymers [86,87] as a function of 1/*M*_n,PSt_ together with the *T*_g_ data versus 1/*M*_c_ of the PSt crosslinked by MA-PDMS-MA. This plot also includes *T*_g_ data reported previously for PSt-*l*-PDMS conetworks [71]. As can be seen in this figure, the *T*_g_ data versus 1/*M*_n,PSt_ for the PSt homopolymers and the *T*_g_ for the PSt in the conetworks as a function of 1/*M*_c_ fit well in the same linear, Fox–Flory-type relationship in this joint plot. This result conclusively verifies the scissors effect of the PDMS macrocrosslinker in the PSt-*l*-PDMS conetworks.

**Figure 5:**
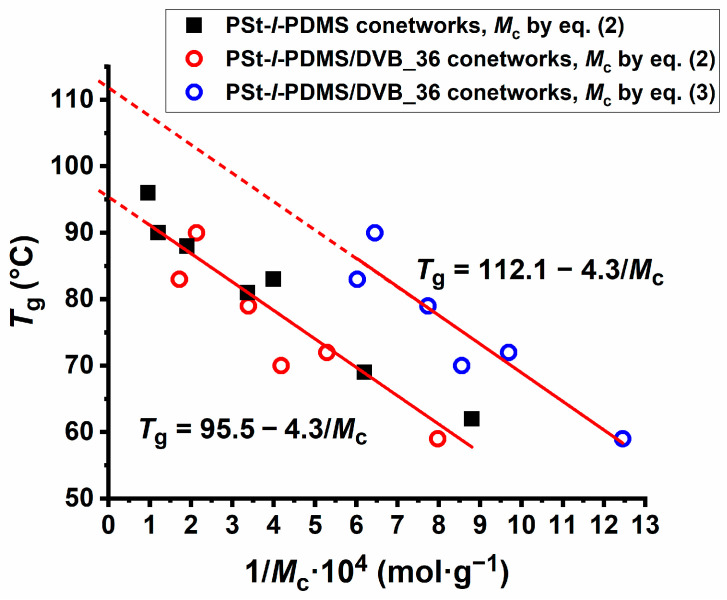
The glass transition temperature of the PSt component as a function of 1/*M*_c_ in the PSt-*l*-PDMS (black square) and PSt-*l*-PDMS/DVB (red and blue circles) conetworks with *M*_c_ values determined by Equation (3) (blue circle) and Equation (2) (red circle and black square).

As displayed in Figure 2B,D, the *T*_g_ of PSt in the PSt-*l*-PDMS/DVB conetworks exhibits a similar trend to that in the PSt-*l*-PDMS conetworks, that is, the *T*_g_ decreases with the increasing PDMS macrocrosslinker content by keeping the DVB/St ratio constant at 1/36 *m*/*m*. Figure 5 shows the *T*_g_ data as a function of 1/*M*_c_, where *M*_c_ is obtained by equation (3). This plot indicates a linear relationship with an intercept of 112.1 °C, which is significantly higher than that of the *T*_g_ of polystyrene with high molecular weight. On the other hand, the slope of the fitted straight line is 4.3 × 10^−4^ °C·g/mol, which is close to the value of the slope of the *T*_g_ versus 1/*M*_c_ plot for the PSt-*l*-PDMS conetworks prepared without DVB, i.e., 4.1 × 10^−4^ °C·g/mol. This indicates that the two plots are nearly parallel to each other. This led us to calculate the *M*_c_ for the PSt-*l*-PDMS/DVB conetworks by taking into account only the PDMS macrocrosslinker, i.e., by neglecting the DVB, according to Equation (2), and to plot the *T*_g_ data as a function of 1/*M*_c_. The left-hand side plot in Figure 5 includes these data together with that of the PSt-*l*-PDMS conetworks. As can be seen, the two kinds of data overlap, indicating that the determining component for the *T*_g_ of PSt in the conetworks is the amount of the MA-PDMS-MA macrocrosslinker. The DVB, with the applied amount, i.e., DVB/St ratio of 1/36 *m*/*m*, does not have a detectable effect on the glass transition behavior in the PSt-*l*-PDMS/DVB conetworks.

**Figure 6:**
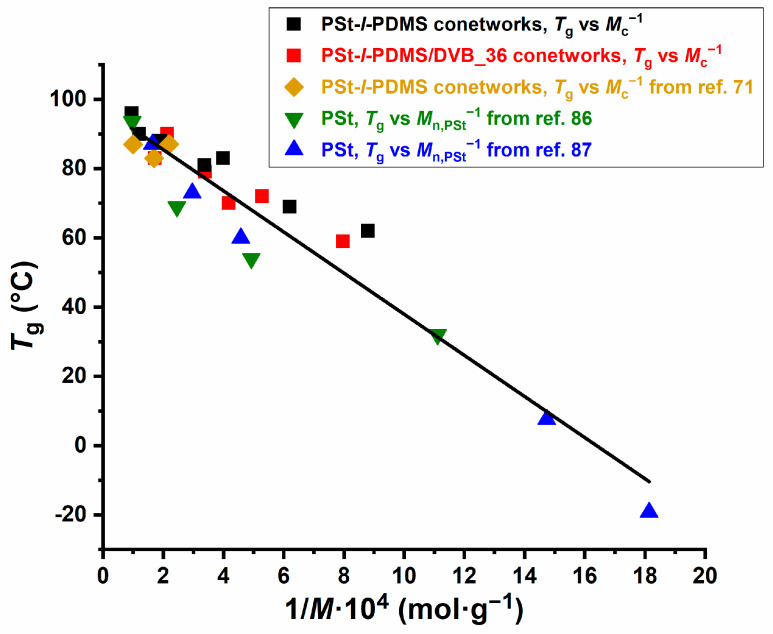
The *T*_g_ of the PSt component of PSt-*l*-PDMS (black and light brown), PSt-*l*-PDMS/DVB (red) conetworks as a function 1/*M*_c_, and the *T*_g_ of PSt homopolymers as a function 1/*M*_n,PSt_ (green and blue). Data from Refs. [71,86,87].

**Scheme 3:**
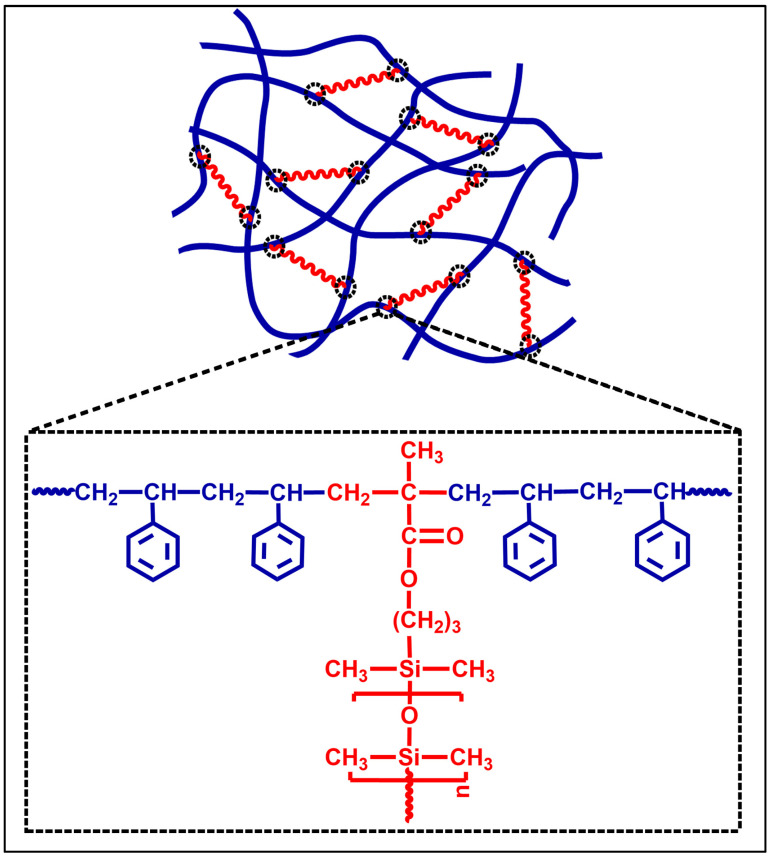
The structure of the crosslinking point in PSt-*l*-PDMS conetworks which interrupts the chain mobility of the PSt segments in these macromolecular assemblies and results in the scissors effect by the PDMS macrocrosslinker on the glass transition behavior of the polystyrene component.

Figure 6 summarizes the observations presented for the Fox–Flory-type correlation between the *T*_g_ and 1/*M*_c_ for the PSt-*l* PDMS and the PSt-*l*-PDMS/DVB conetworks and the *T*_g_ and *M*_n_ literature data for polystyrene homopolymers [86,87]. As this plot indicates, these data show that both the 1/*M*_c_ and 1/*M*_n,PSt_ dependence for the conetworks and PSt homopolymers, respectively, exhibits the same trend, validating the scissors effect of the PDMS macrocrosslinker on the glass transition behavior of PSt in the conetworks. These findings indicate that the junction points between the PDMS macrocrosslinker and the crosslinked PSt chains significantly interrupt the mobility of the PSt chains, as illustrated in Scheme 3, by displaying the structure of the junction points in the conetworks. The difference in the chain mobility between the PDMS macrocrosslinker and the crosslinked PSt chains leads to chain segments of PSt with molecular weight of *M*_c_ having the same mobility, and thus, glass transition temperature as PSt homopolymers with the same *M*_n,PSt_. As a result, the *T*_g_ versus 1/*M*_c_ plot shows Fox–Flory-type dependence. This explanation of the scissors effect by the macrocrosslinker is supported by the results of previous solid-state NMR experiments with poly(N,N-dimethylaminoethyl methacrylate)-*l*-polyisobutylene (PDMAEMA-*l*-PIB) conetworks prepared with deuterated methacrylate-ended PIB [92]. As found in this study, the more mobile PIB macrocrosslinker with lower *T*_g_ significantly influences the mobility of the crosslinking point, and this results in lower *T*_g_ of the PDMAEMA component than that of its homopolymer, similar to the investigated PSt-*l* PDMS and PSt-*l*-PDMS/DVB conetworks.

## 4. Conclusions

In order to explore the relationship between the *T*_g_ and *M*_c_ in PSt-*l*-PDMS and PSt-*l*-PDMS/DVB conetworks, a library of these conetworks, consisting of two immiscible, covalently bonded polymers, i.e., PSt and PDMS, was prepared with a wide composition range of 30–80 wt% PDMS content. For the synthesis, MA-PDMS-MA was used as a macrocrosslinker with varying *M*_n,PDMS_ of 4.7, 9, and 22.2 kDa. In the presence of DVB, the St/DVB weight ratio was 36:1 when DVB was present as an additional low molecular weight crosslinker. The results of DSC measurements revealed two separate glass transition temperatures, that is, one for the PDMS component in the range of the PDMS homopolymers at around −120 °C, and another for PSt in the range of 60−100 °C. This indicates strong segregation of PSt and PDMS in these conetworks, in agreement with previously published SAXS and AFM results on the nanophase-separated morphology of these conetworks [73]. The DSC thermograms show that the crystallization of the PDMS macrocrosslinkers with *M*_n,PDMS_ of 4.7 and 9 kDa is completely suppressed in the conetworks. This is due to the nanoconfinement of the PDMS nanodomains, which prevents nucleation for crystallization in such conetworks. In the case of conetworks with PDMS having higher *M*_n,PDMS_ (22.2 kDa), i.e., with longer chains, depleted cold crystallization and melting peaks appear in the DSC scans. Surprisingly, the *T*_g_s of the PSt component in both the PSt-*l*-PDMS and PSt-*l*-PDMS/DVB conetworks were found to decrease with decreasing *M*_c_—that is, with increasing the PDMS content. Considering the well-known increase in *T*_g_ with decreasing *M*_c_ in the cases of conventional polymer networks crosslinked with low molecular weight crosslinkers [75,76,77], correlation analysis was carried out for the unusual *M*_c_ dependence of *T*_g_ of the crosslinked PSt in the conetworks. It was found that the *T*_g_ as a function of *M*_c_ follows the Fox–Flory relationship [74], which is obtained for the dependence of *T*_g_ on *M*_n,PSt_ for homopolymers. This unexpected finding indicates that the MA-PDMS-MA macrocrosslinker behaves like scissors in these conetworks in relation to the glass transition of the crosslinked PSt chains, leading to *T*_g_s which matches that of the PSt homopolymers with the same *M*_n,PSt_ as *M*_c_. In other words, the *T*_g_ of the PSt component is determined by the scissors effect of the PDMS macrocrosslinkers in these conetworks. On the basis of a literature result, according to which the mobility of the molecular segment of the crosslinking point is similar to that of the macrocrosslinker in the PDMAEMA-*l*-PIB conetwork [92], it can be concluded that the scissors effect is due to this difference in mobility at and/or most likely in the close vicinity of the crosslinking structural units. Taking into account the findings in this study and similar previous observations with other conetworks [12,13], it can be claimed that the scissors effect can be regarded as a general phenomenon for conetworks prepared by using macromolecular crosslinkers for conetwork formation. The existence of the scissors effect enables designing and applying polymer conetworks with predetermined *T*_g_s for the crosslinked polymer components in such macromolecular assemblies by selecting the proper *M*_c_ via either composition or by the *M*_n_ of the applied macromolecular crosslinker.

## Figures and Tables

**Table 1:** The gel fraction, composition (PDMS content), the average molecular weight between crosslinks (*M*_c_), and the glass transition temperature (*T*_g_) of the PSt component of PSt-*l*-PDMS and PSt-*l*-PDMS/DVB conetworks.

Sample ID	Gel Fraction (%)	PDMS (wt%)	*M*_c_ (Equation (2)) (g/mol)	*M*_c_ (Equation (3)) (g/mol)	*T*_g_ (°C)
S-4.7-48	55.9	48.4	2510	-	83
S-4.7-59	60.4	59.3	1610	-	69
S-4.7-67	60.2	67.4	1140	-	62
S-4.7-76	63.0	76.4	730	-	n.d.
S-4.7-84	53.2	84.1	440	-	n.d.
S-22.2-51	41.3	51.4	10500	-	96
S-22.2-57	59.6	57.2	8310	-	90
S-22.2-68	54.1	67.9	5250	-	88
S-22.2-79	61.1	78.9	2970	-	81
S-22.2-89	59.6	89.0	1370	-	n.d.
SD36-4.7-33	88.1	33.4	4690	1550	90
SD36-4.7-44	86.3	44.3	2960	1290	79
SD36-4.7-55	86.7	55.4	1890	1030	72
SD36-4.7-65	87.1	65.2	1250	800	59
SD36-4.7-75	86.8	75.4	770	570	n.d.
SD36-9-65	94.6	65.3	2390	1170	70
SD36-22.2-66	91.1	65.6	5820	1660	83

n.d.: not detectable.

## Data Availability

The original contributions presented in this study are included in the article. Further inquiries can be directed to the corresponding author.
